# A Cytochrome P450 Facilitates Polyethylene Metabolism in a Microbial Community

**DOI:** 10.3390/ijms26188775

**Published:** 2025-09-09

**Authors:** Madelyn Tarara, Shivani Ahuja, Jay L. Mellies

**Affiliations:** 1 Department of Biology, Reed College, Portland, OR 97202, USA; m.tarara@utah.edu; 2 Department of Chemistry, Reed College, Portland, OR 97202, USA; ahujas@reed.edu

**Keywords:** low-density polyethylene, PE plastic, biocatalyst, depolymerization, biodegradation, cytochrome P450 reductase, CYP, bacterial consortium

## Abstract

The synthetic polymer low-density polyethylene (LDPE) is a pervasive pollutant that poses serious environmental concerns and health hazards. PE plastic is rarely recycled, and therefore, biodegradation is a novel approach for managing PE plastic waste. However, few enzymes and organisms that degrade PE plastic have been identified to date. Herein, we demonstrate that a consortium of soil bacteria containing *Pseudomonas* and *Bacillus* species can grow on and degrade UV-treated PE film and powder as a sole carbon source, reducing its net mass by 7% and 13%, respectively, in 30 days. Changes in surface functional groups associated with chemical modification of PE were observed via ATR-FTIR analysis, and byproducts associated with PE biodegradation and alkane and carboxylic acid metabolism were observed via GC/MS. Using previously characterized PEases, we identified a gene, CYP102 A5, found in *Bacillus thuringiensis* strain 9.1, which encodes a cytochrome P450 reductase, whose expression was increased when grown on PE as a sole carbon source. Purified CYP102 A5 protein altered PE surface functional groups, determined by ATR-FTIR, giving evidence of PE oxidation. In sum, we identified a cytochrome P450 reductase that explains, in part, how a consortium of soil bacteria can grow on and degrade PE plastic.

## 1. Introduction

Plastic pollution is a growing crisis facing our planet. A large contributor to our global waste problem is low-density polyethylene (LDPE), a specific type of polyethylene (PE) plastic waste. PE plastics have meager recycling rates; in the U.S, 8.59 million tons of LDPE/linear low-density polyethylene (LLDPE) were produced in 2018, yet only 4.3% of this plastic was recycled in municipal waste streams in that year [1]. On a global scale, the production of LDPE is estimated to be around 17.4% of the total international production market due to its widespread use in agriculture, packaging, and construction [2,3,4].

PE pollution has detrimental effects on our ecosystems, particularly within marine environments—it is estimated that out of the total 460 million tons of plastic produced every year, 9−14 million tons end up in our oceans [5,6]. The same chemical properties that make plastics attractive for commercial use, i.e., non-conductive and chemically inert, enable plastics to persist in the environment, contributing to long-lasting, negative environmental impacts. Namely, in aquatic environments, plastics can break down into microplastics (5 mm–1 nm in size) and eventually into smaller nanoplastics (1 nm–1 µm in size). PE microplastics are formed when these products are exposed to solar radiation and to physical and mechanical forces. PE microplastic exposure is implicated in negative effects on multiple animals. For example, they affect gene expression, and on the larger scale, cognition and behaviors of aquatic organisms, like the planktivorous Indo-Pacific pelagic fish *Decapterus muroadsi*, which was found to eat microplastics that resemble their prey, weakening already fragile ecosystems [7]. Polyethylene exposure increases mortality and decreases locomotion in *Collembola* hexapods, which contribute to ecosystems by shaping soil microbial communities, and *Apis mellifera* bees, the most prevalent honey bee species, which pollinate crops and various other plants [8,9]. Studies are beginning to elucidate the negative health impacts of PE microplastics in both mouse models and in human cells, including developmental/reproductive toxicity, cytotoxicity, and chromosomal instabilities. Microplastics can cause particle toxicity, which has been shown to induce immune responses, as well as chemical toxicity from incorporated additives and absorbed environmental pollutants [10,11,12,13].

The properties of PE limit recycling due to the strong C-C and C-H bonds, which are difficult to break [14,15]. The carbon backbone has no additional functional groups and is therefore inert and resistant to degradation [16]. Besides conventional recycling, other methods, including thermal and chemical recycling, are known to be hazardous to the environment. Incineration of PE can cause air pollution via emissions of greenhouse gases and produce furans, carbon monoxide, dioxins, and other toxic compounds which cause negative downstream effects on native flora and fauna [17,18].

Given the cost to environmental and human health associated with other recycling methods, biodegradation of PE is gaining interest. A variety of microbes grow on PE, including *Pseudomonas aeruginosa*, *Rhodococus ruber*, *Bacillus cereus*, and *Bacillus thuringiensis* [19,20,21,22,23,24]. Microorganisms that can degrade PE often have slow growth and degradation rates, ranging from 5−20% weight loss of PE after 60 days of incubation [19,25,26,27]. The high crystallinity, hydrophobicity, and high molecular weight of PE limit the efficiency of microbial biodegradation [28,29]. An improvement on the strategy of using single microbial isolates is to instead use microbial consortia, or groups of bacteria, that work synergistically, which may improve degradation rates due to greater metabolic versatility [30].

The ability for bacteria to utilize LDPE as a carbon source has been described, but only four enzymes have been cataloged on the open-source enzyme database “Plastics DB” to date [31]. However, a diverse array of enzymes, including lignin peroxidases, manganese peroxidases, and laccases, degrade oxidizable C-C bonds [32,33]. Additionally, alkane hydrolases and cytochrome P450 s degrade LDPE via terminal or subterminal carbon oxidation [34]. Researchers have suggested that PE is depolymerized by extracellular enzymes, and then the byproducts are brought into the cell [35], where mineralization can occur. Due to the diversity of enzymes present in hydrocarbon metabolism, it is vital to fully characterize the mechanisms necessary to degrade PE. Characterization of enzymes will enable the development of a model mechanism for LDPE degradation, and in parallel, provide insights into how to engineer the process for better efficiency, by upregulation of key conversion steps and regulatory elements, incorporating more efficient engineered enzymes or introducing enzymes from other species [35].

We previously isolated a consortium of three *Pseudomonas* and two *Bacillus* strains that degrade PET plastic [36]. Analyzing the metagenomes, we found 250 genes encoding enzymes associated with the degradation of synthetic plastics, including PE, biopolymers, and plasticizers [37]. In this report, we demonstrate that the five-member consortium of bacteria isolated from petroleum-polluted soil can grow on and synergistically degrade PE plastic, up to 13% weight reduction after a 30-day incubation. Surface modification of the PE was observed by ATR-FTIR, and degradation byproducts were identified by GC/MS. We used bioinformatics and RT-qPCR to identify putative PEases with differential expression when grown on PE. The gene encoding CYP102 A5 [24], a cytochrome P450 reductase of *Bacillus thuringiensis*, showed the greatest increased transcription when grown on PE, and the purified enzyme altered surface functional groups, indicating oxidation of the material. Thus, we observed the growth of a bacterial consortium on PE plastic and identified a P450 enzyme that most likely initiates biodegradation.

## 2. Results

### 2.1. Bacterial Consortium Uses PE as a Sole Carbon Source

We grew the bacteria on PE as a sole carbon source in LCFBM to determine whether the full consortium can degrade PE plastic. Growth curves are plotted in Figure S1. Cultures enriched with UV-treated PE plastic and supplementary yeast extract, to initiate growth, were compared to cultures without bacteria to ensure no contamination occurred and to control for any spontaneous PE degradation that might occur within the sterile media. The growth curve was monitored over 30 days to evaluate bacterial colonization and growth.

**Figure 1:**
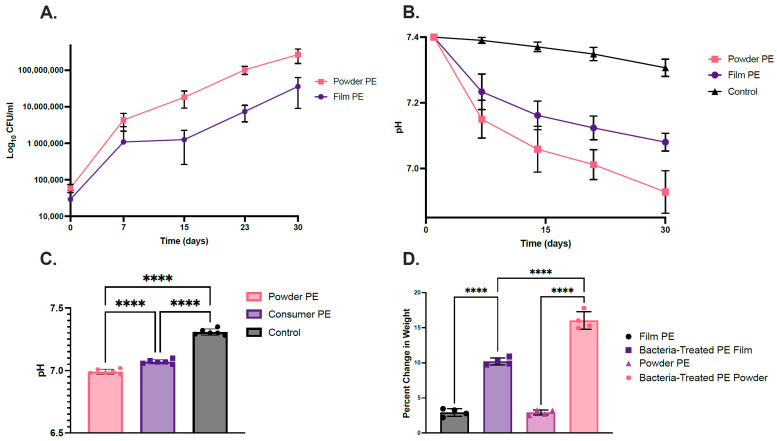
(**A**) Growth curves of the bacterial full consortium in LCFBM in two PE plastic sources: PE powder and PE film for 30 days. (**B**) pH change over time of the LCFBM medium inoculated by the bacterial full consortium over 30 days. (**C**) pH change after 30 days of the LCFBM medium inoculated by the bacterial consortium. (**D**) Percent change of plastic weight of pure PE film and powder and degraded PE powder cultured with the bacterial consortium. Untreated pure powdered PE is the control group (**** *p* ≤ 0.0001).

CFU/mL values indicated that the bacterial consortium grew on both PE plastic sources (0.2% *w*/*v*) over the 30-day culture time (Figure 1A). The powdered PE culture grew to a CFU/mL of 2.6 × 10^8^, whereas the PE film culture grew to a CFU/mL of 3.58 × 10^7^. This agrees with other studies showing that a higher surface area to volume ratio for the PE powder promotes better biofilm formation and therefore higher capacity for growth [38]. The control had no bacterial growth for both plastic types. These results are consistent with the observation that both bacterial-treated plastic substrates had a statistically significant decrease in pH after 30 days compared to the control (*p* ≤ 0.0001) (Figure 1B). The bacteria-treated powder PE exhibited a greater decrease in pH compared to the culture with PE film (*p* ≤ 0.0001). Bacterial growth and pH changes, indicating metabolic activity producing acidic byproducts, suggested that the bacteria were metabolizing the PE plastic. The control had only a slight decrease in pH change from the initial 7.4 compared to media containing bacteria (Figure 1B). Figure 1C shows that the bacteria-treated PE powder and film had an average percent weight loss of ~16% and ~10%, respectively, when normalized against natural hydrolysis in the control (*p* < 0.0001). Due to the increase in bacterial CFU/mL in media containing polyethylene, the change in pH values, and plastic weight loss over time, we concluded that the bacterial consortium can utilize PE plastic as a carbon source.

### 2.2. Surface Modifications of PE Plastics After Degradation

To determine whether the PE surface was being degraded, we first removed the bacteria by washing with 2% SDS and then observed the surface of amorphous PET using scanning electron microscopy (SEM). Bacteria utilize enzymes like laccase or manganese peroxidase, which often catalyze plastic biodegradation via depolymerization. Oxidation generally occurs from an edge and increases the roughness of the plastic surface [39].

**Figure 2:**
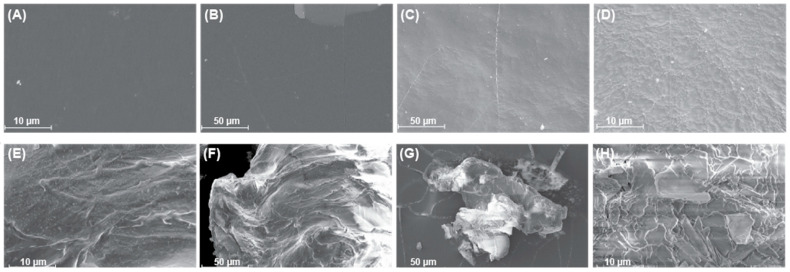
SEM images show the surface modification of PE film and powdered PE. LCFBM cultures containing either film PE (**A**,**B**,**E**,**F**) or PE powder (**C**,**D**,**G**,**H**) and the full consortium were grown at 30 °C for 30 days. Bacteria and biofilms were removed from the surface using 2% SDS and washed with sterile water and 70% ethanol to remove any cellular debris. Images (**A**,**B**) depict PE film untreated with bacteria, and images (**D**,**F**) show PE film treated with bacteria. Images (**C**,**D**) show powdered PE not treated with bacteria, and images (**G**,**H**) show plastic treated with the full consortium.

The structural changes in both PE film (Figure 2A,B,E,F) and PE powder (Figure 2C,D,G,H) showed increased roughness on the surface in bacteria-treated PE samples when compared to the untreated control. The higher degree of roughness observed on the powdered PE samples versus the film samples is enforced by the greater surface area to volume ratio, thus, more efficient enzymatic degradation due to increased area that biofilms can form and degrade PE [40,41]. The difference in roughness of the surfaces, comparing the bacterial-treated PE and natural hydrolysis of the control, was attributed to bacterial growth on the plastic surface.

**Figure 3:**
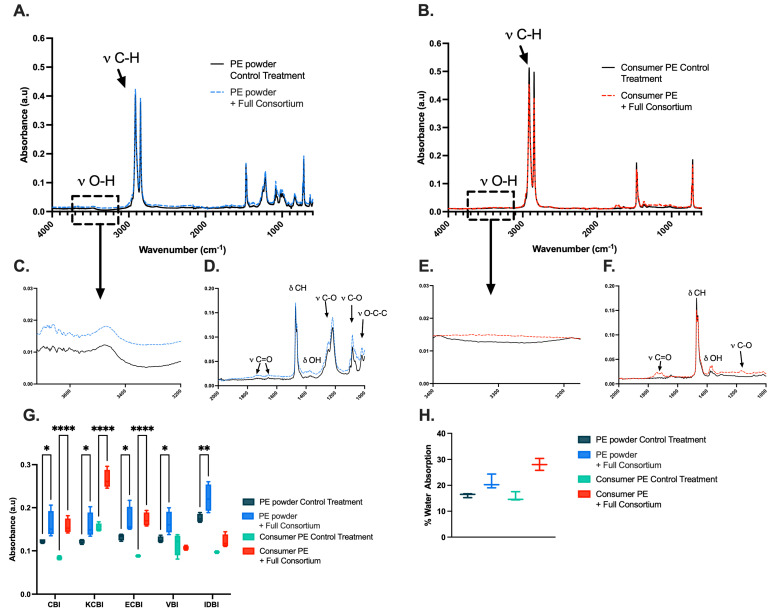
(**A**) ATR-FTIR spectra of average untreated pure PE powder (*n* = 3) and averaged PE film (*n* = 3) treated with the bacterial consortium. (**B**) FTIR-ATR spectra of average pure PE powder (*n* = 3) and average PE powder (*n* = 3) treated with the bacterial consortia. (**C**) Inset of OH stretching of PE powder with and without treatment of the bacterial consortia. (**D**) Inset of carbonyl and ester stretching of PE powder with and without treatment of the bacterial consortia. (**E**) Inset of OH stretching of consumer PE with and without treatment of the bacterial consortia. (**F**) Inset of carbonyl and ester stretching of consumer PE with and without treatment of the bacterial consortia. (**G**) Means of carbonyl indexes (*n* = 3) of PE film and powder treated with the full consortium and associated film and powder controls (CBI—Carbonyl Bond Index; KCBI—Keto Carbonyl Bond Index; ECBI—Ester Carbonyl Index; VBI—Vinyl Bond Index; IDBI—Internal Double Bond Index; * *p* ≤ 0.05, ** *p* ≤ 0.01, **** *p* ≤ 0.001). (**H**) Percent Water Absorption of PE powder and film with and without treatment of the bacterial consortium.

We examined changes in the chemical structure of the PE plastic using ATR-FTIR analysis. Both types of PE samples treated by the bacterial consortium exhibited changes in peaks associated with functional group bending and stretching. The PE film (Figure 3A) exhibited ATR-FTIR peaks at 3315 cm^−1^, 1366 cm^−1^, 1709 cm^−1^, 1418 cm^−1^, and 1220 cm^−1^, corresponding to -OH stretching and bending (hydroxyl group) [42,43,44], C=O stretching (carboxyl groups, hydroxyl group) [45,46], and C-O stretching (ester) [47]. Additionally, characteristic peaks at 1742 cm^−1^ were attributed to C=O bending (aldehyde) [48]. The PE powder (Figure 3B) exhibited similar ATR-FTIR peaks at 3315 cm^−1^, 1369 cm^−1^, 1709 cm^−1^, 1406 cm^−1^, and 1219 cm^−1^. The PE powder (Figure 3B) did not have any significant differences in 1740−1720 cm^−1^ compared to the control. However, changes were observed at 670 cm^−1^, indicating C=C bending (alkene) [49].

Changes in various absorbance intensities of relevant bond wavelengths when compared to the methylene bond were observed after 30 days of incubation with the bacterial consortium for both PE powder and film (Figure 3G, Table 1). When comparing the bacteria-treated samples to the control PE plastics, most indices exhibited statistical significance. The PE film exhibited high statistical significance for the carbonyl bond index (CBI), keto carbonyl index (KCBI), and the ester carbonyl bond Index (ECBI) (*p* < 0.0001). Treated PE powder also exhibited statistical significance for the CBI (*p* = 0.0266), KCBI (*p* = 0.0364), and the ECBI (*p* = 0.0224), although to a lesser extent than the film treatment group. However, the vinyl bond index (VBI) and the internal double bond index (IDBI) varied between the powder and film, with statistical significance observed in the powder group (*p* = 0.0223 and *p* = 0.004, respectively) while the film group displayed no significant difference in these same indices *(p* = 0.9009 and *p* = 0.2397).

The change in water absorption for PE film and PE sheets was also calculated (Figure 3H). When comparing the PE film culture to its control, it approached statistical significance (*p* = 0.0588), while the PE powder was significant (*p* = 0.0004) when compared to the samples not treated with bacteria. In short, we concluded that there were various new functional groups in bacteria-treated PE samples compared to controls, supporting our previous conclusions that the bacterial consortium can metabolize PE. Additionally, we concluded that the PE powder cultures exhibited greater changes in surface functional groups and total water absorption after bacterial treatment.

### 2.3. Byproducts Resulting from Bacterial Degradation of PE Plastic

**Figure 4:**
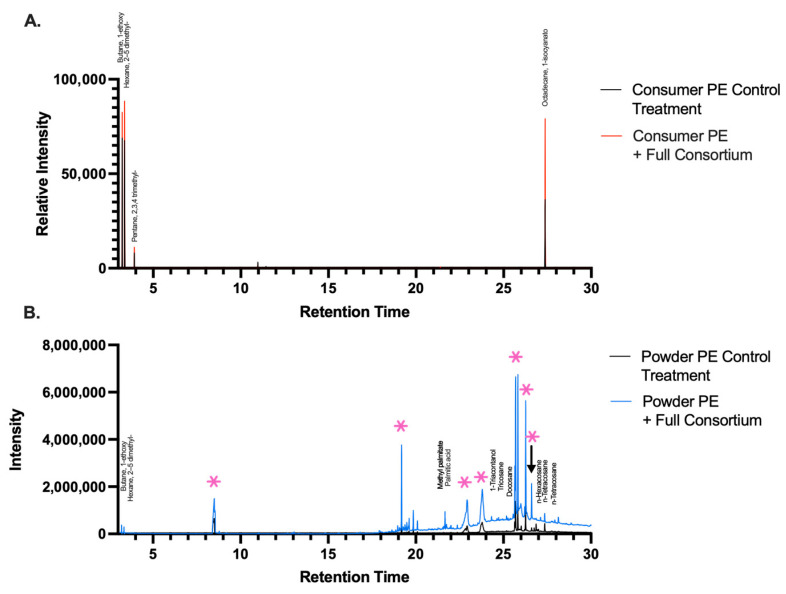
(**A**) GC/MS chromatogram of averaged relative intensity (*n* = 4) normalized to the blank of pure PE film and degraded PE film cultured with the full consortium of metabolites found in the bacterial supernatant. Untreated film is the comparative control. (**B**) GC/MS chromatogram of averaged relative intensity (*n* = 4) normalized to the blank of pure PE powder and degraded PE powder cultured with the full consortium. Hydrolysis of PE powder is the control group. Compounds associated with plastic are indicated via a pink asterisk, from left to right: diethylene glycol, azulene, 1,4-dimethyl−7-(1-methylethyl), two unknown phenolic compounds, 2,4-bis(1-phenylethyl)phenol, diethylene glycol dibenzoate, and dioctyl phthalate. (See also Table S2).

After identification of functional groups on the surface of the PE plastic, we performed GC/MS analysis of bacterial culture supernatants to characterize the metabolites formed after bacterial degradation of PE. GC/MS results revealed the formation of several products: alkanes, aldehydes, and plastic additives (Figure 4A; Tables S1 and S2). The PE powder culture showed the formation of a wider range of alkanes, ethers, aldehydes, and carboxylic acid compounds during the degradation process (Figure 4B). Both plastic samples, comparing bacterial treatment with spontaneous hydrolysis, identified compounds that are common plastic additives. Our GC/MS results were consistent with the decomposition of PE being broken down to lower molecular weight monomers [24,44,50].

### 2.4. Identification of Enzymes Putatively Associated with PE Metabolism

We used a bioinformatic approach to identify enzymes involved in PE metabolism. We putatively identified four enzymes with PE-degradation capabilities, using a sequence similarity greater than 25% to known PEases: a cytochrome P450 NADPH reductase (98% similarity) from *B. thuringiensis* (9.1), a glutathione dehydrogenase (GlutD) in *Rhodococcus* sp. C−2 with 78% similarity with our two *Pseudomonas* sp. strains (10 and 13.2). A Baeyer-Villager monooxygenase (BVMO) isolated from *Alcanivorax* sp. 24 was 25.64% similar to *Pseudomonas* B10 (9.2), and the carboxylesterase NlhH identified in *Alcanivorax* sp. 24, had a 38.79% similarity to *Pseudomonas* strains 10 and 13.2, and was 32% similar to that in strain 9.2. After identification of these genes, we utilized reverse-transcription quantitative PCR (RT-qPCR) (Table S3) to test for induced expression when grown in culture with PE plastic.

**Figure 5:**
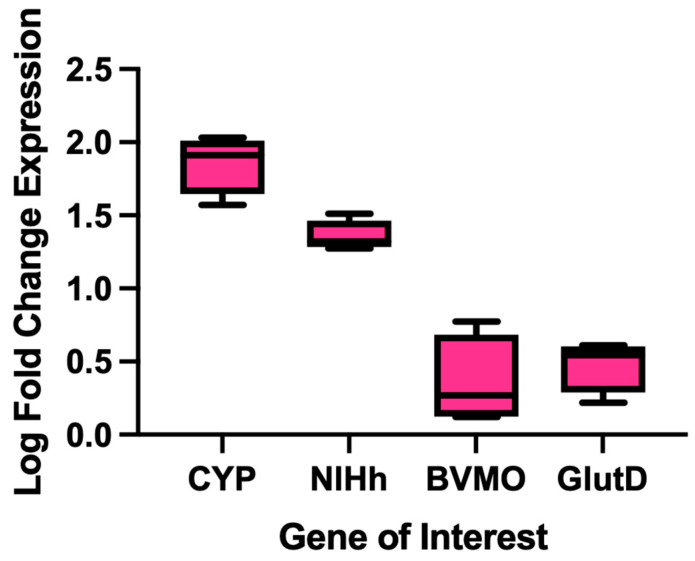
RT-qPCR expression data of genes of interest for individual strains when grown on PE plastic. Log fold change expression determined by two-step quantitative real-time PCR (RT-qPCR) of genes putatively involved in PE biodegradation. Fold change expression was calculated (*n* = 4) using the Pfaffl method for bacterial isolates. Isolate associated with each tested gene, and the associated primer sequences are described in Supplementary Table S3.

RT-qPCR analysis was performed using the 16 s rRNA gene as the internal control. We measured transcriptional activity for the putative PE-degrading genes: *cyp* (strain 9.1), BVMO (strain 9.2), *glutD* (strain 10), and *nlhH* (strain 13.2). Expression of the BVMO and *glutD* genes was not significantly different under the conditions tested (*p* = 0.7504 and 0.3077). The *nlhH* gene was slightly upregulated in the presence of PE plastic (1.356-log fold; *p* = 0.002). CYP expression was greater than that of *nlhH*, a 1.857 log fold change (*p* < 0.0001) (Figure 5). We thus hypothesized that the enzymes NlhH and CYP may be associated with PE metabolism. It is important to note, however, that the genomes of the five axenic strains are diverse and encode many more hydroxylases, dehydrogenases, hydrolases, and monooxygenases [37] that may participate in PE metabolism, but were not examined using RT-qPCR.

### 2.5. Purified CYP102 A5 Cytochrome P450 Reductase Enzymatic Activity in the Presence of PE Plastic

**Figure 6:**
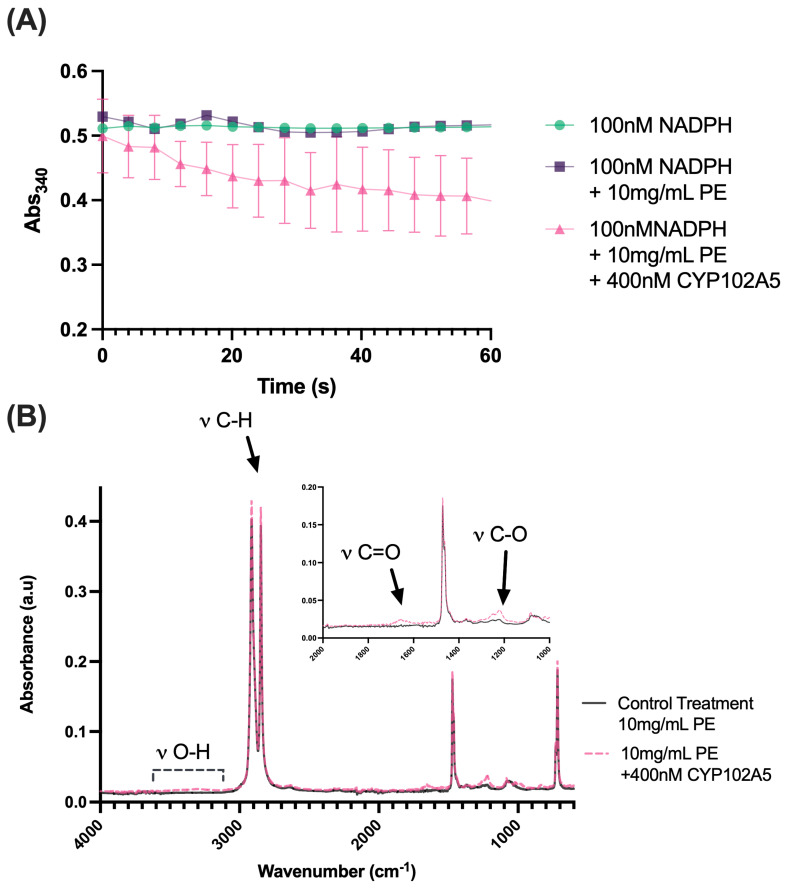
(**A**) NADPH consumption by CYP102 A5 in the presence of 10 mg/mL powdered PE plastic over 60 s (*n* = 6) with 95% CI intervals plotted. Representative controls of NADPH in buffer (*n* = 4) and NADPH and 10 mg/mL PE (*n* = 4) are also presented. (**B**) Averaged ATR-FTIR data of PE powder either with or without treatment with CYP102 A5 for 18 h (*n* = 8).

To determine whether increased mRNA expression levels correlated with CYP102 A5 modifying PE, we measured the catalytic activity of CYP102 A5 using PE as a substrate. We observed an oxidation rate of 23.55 nmol NADP^+^ min^−1^ (nmol of P450^−1^) (Figure 6A)**.** New peaks were found in the enzyme-treated samples by ATR-FTIR (Figure 6B) the presence of OH stretching in the polymer between 3350 cm^−1^ and 3200 cm^−1^, a new C=O stretching peak was observed between 1702 cm^−1^, and two new C-O stretching peaks were identified at 1252 cm^−1^ and 1090 cm^−1^ (Table 2).

## 3. Discussion

In this study, we demonstrate that a bacterial consortium consisting of three *Pseudomonas* and two *Bacillus* strains isolated from petroleum-polluted soil grows on PE plastic. The bacteria degrade the PE relatively efficiently; we observed a 16% weight loss for PE powder and a 10% weight loss for PE film after 30 days. Increased bacterial populations combined with a decrease in pH of the medium suggested depolymerization and fragmentation of PE into lower molecular weight compounds, which was demonstrated by ATR-FTIR [51,52]. These results together demonstrate that the bacterial consortium grows on PE as a sole carbon source. The description of microbial consortia degrading PE plastic has been limited to date.

Bacteria form biofilms on hydrophobic plastic surfaces, which are crucial for PE degradation [53]. Biofilm matrices enable plastic degradation through the secretion of exopolysaccharides for adhesion, and enzymes, such as laccases, hydrolases, and esterases, which facilitate the breakdown of the polymer [54,55,56,57]. As polymer biodegradation proceeds, the surface characteristics are altered, which can be observed via ATR-FTIR. We observed distinct changes in carbonyl indices for both PE powder and film, suggesting that the bacterial community oxidizes the surface of PE, while the appearance of new peaks, such as alcohols, ethers, and alkanes (Tables S1 and S2) indicates further degradation mediated by other secreted enzymes during depolymerization [58]. Higher water absorption (Figure 3D) means the polymer is becoming more hydrophilic, indicating that the bacterial consortia make the surface more hydrophilic.

We identified several metabolic byproducts by GC/MS after a 30-day degradation period, primarily alkanes, but also alkanes with carboxylic acid, aldehyde, and alcohol functional groups (Tables S1 and S2). These compounds are consistent with what is currently known of PE degradation and the enzymatic diversity of the degradative process [59,60]. The degradative byproducts often resemble fatty acids, which are eventually imported into the bacterium and catabolized via β-oxidation. Small molecules are converted into acetyl-CoA and enter the tricarboxylic acid (TCA) cycle and are finally mineralized via respiration. Identifying the metabolic byproducts was a focus of this work because it would suggest the types of enzymatic reactions occurring during degradation. Overall, the byproducts identified in the GC/MS analysis, combined with the changes in surface functional groups observed through ATR-FTIR, corroborate our previous findings that the bacteria are biodegrading PE and assimilating PE byproducts.

Four enzymes with >25% identity to previously characterized PE enzymes were evaluated via RT-qPCR. Of these four enzymes, a bi-functional cytochrome P450 NADPH reductase, encoded in *Bacillus thuringiensis* strain 9.1, was upregulated to 1.73 log fold when grown on PE in isolation and increased to 2.76 log fold when grown in combination with the full consortium on PE (Figure S2). This cytochrome P450 shares 99% sequence similarity with CYP102 A5.v1, which was solved to 0.93 Å and was previously shown to degrade PE plastic [24], and was thus named CYP102 A5 (Supplemental Text S2). CYP102 A5.v2 possesses the following mutations: S229 N, D231 N, P468 S, T673 I, K822 R, and M941 I (Supplemental Text S3 and Figure S5), several of which were previously targeted for engineering the closely related CYP102 A1/BM3 enzyme to improve degradation of n-alkanes [61,62,63,64]. CYP102 A5 is natively involved in combating polyunsaturated fatty acid toxicity and catalyzes rapid substrate oxidation (compared to turnover rates from other P450 monooxygenases) at various ω hydroxylation positions [65]. Therefore, increased water absorption/hydrophilicity of the PE caused by CYP102 A5.v2 was predicted to occur by enzymatic biodegradation via ω hydroxylation and enzymatic activity through the consumption of NADPH (Figure 6). When using PE as a substrate, we measured the catalytic activity of CYP102 A5.v2 and found a rate of 23.55 nmol NADP+ min^−1^ (nmol of P450)^−1^, while CYP102 A5.v1 for NADPH oxidation was 8.588 nmol of NADP+ min^−1^ (nmol of P450^−1^). These data, paired with the observed mutations, bolster the evidence that CYP102 A5.v2 is directly involved in PE oxidation.

However, the specific mechanism of CYP102 A5.v2-mediated PE degradation in *Bacillus thuringiensis* requires further investigation. CYP102 A5.v2 is a relatively large enzyme, with the predicted dimer being ~240 kDa (Figure S3), and it lacks a signal sequence for secretion, which can be predicted using SignalP 6.0 software. Thus, the enzyme is likely not secreted from *B. thuringiensis* via conventional secretion systems and necessitates more research [66].

The cytochrome P450 reductase (CYP) facilitates terminal, subterminal, and in-chain hydroxylation of alkanes [35]. Baeyer–Villiger Monooxygenases (BVMOs) convert in-chain or subterminal ketones to esters and oxidize aldehydes [67,68]. This might explain the presence of palmitic acid, which contains a formate group (Figure S4). Glutathione dehydrogenase was identified as an alcohol dehydrogenase [69], which converts alcohols into ketones, regardless of position on the alkane chain [70]. While we confirmed one enzyme, CYP102 A5.v2, that hydroxylates PE plastic, evidence exists indicating that other enzymes that oxidize PE are found in the consortium pangenome [36,37], and the complete degradation pathway(s) remain to be determined.

Microbial consortia are advantageous for breaking down PE and other types of plastics for multiple reasons. They are more stable than purified enzymes, which require highly purified conditions, and are more stable than single microbial isolates under metabolic stress conditions. They adapt to new environments better and exhibit greater metabolic diversity [71,72]. We have also observed synergistic growth in previous studies on PET plastic, where the full consortium utilized here can reach higher CFU/mL and OD_600_ values, or culture yield, than individual isolates, and thus, are adapting quicker to nutrient-poor plastic environments [73]. With multiple isolates or species, synergy occurs, often through cross-feeding. PE biodegradation can be optimized by identifying additional consortium enzymes that oxidize PE and gaining a comprehensive understanding of the degradation pathways.

Bacterial consortia, like those used in this study, have the potential to significantly reduce the environmental impact of PE plastic waste. Additionally, the metabolic diversity of microbial consortia can lead to valorization, or the identification of valuable chemicals that can be harvested during the biodegradation of PE. Often, artificial intelligence (AI) is used in such efforts because of the complexity of synthetic systems. The waste stream can be reduced through biodegradation. Further, the extraction of compounds like palmitic acid, which is used in the cosmetic industry, methyl palmitate, a major component of biodiesel, or 2,5-dimethyl-hexane, which is used for aviation fuel (Tables S1 and S2), can be targeted. With improved degradation rates and the requisite technology to capture valuable byproducts, recycling of PE would become feasible, a vast improvement over the current handling of the waste, often landfill being the only option. In sum, the sheer mass of PE plastic ending up in landfills could be reduced using environmentally friendly, biological technologies.

## 4. Materials and Methods

### 4.1. Bacterial Strains and Plasmids Utilized in This Study

The five bacterial strains comprising the consortium used for PE plastic degradation in this study were previously obtained from petroleum-polluted soils in Galveston Bay, Texas [36]. Isolated species are *Pseudomonas* sp. SWI36, NRRL B−67630, strain 10; *Bacillus albus* strain PFYN0, NRRL B−67631, strain 13.1; *Bacillus thuringiensis* strain C15, NRRL B−67632, strain 9.1; *Pseudomonas* sp. strain B10, NRRL B−67633, strain 9.2; and *Pseudomonas* sp. SWI36, NRRL B−67634, strain 13.2. Plasmid pMTBN28 cyp was created using the pET28 b(+) expression vector.

For protein overexpression, three commercial strains were used: *E. coli* 10- β (New England Biolabs, Ipswich, MA, USA) for Gibson Assembly, and *E.coli* BL21 DE3 (New England Biolabs, Ipswich, MA, USA) for expression testing, and C41 (DE3) OverExpress™ for expression and purification (Sigma Aldrich, Saint Louis, MO, USA).

### 4.2. Bacterial Growth

#### 4.2.1. Pretreatment of PE Plastic

Polyethylene powder (Sigma Aldrich, Saint Louis, MO, USA) and PE one-sided cling machine film 0.55 mm (Sigma Summit, Belleville, ON, CA) were treated with UV radiation at 365 nm on a UV2 UV Transilluminator (Analytik Jena, Jena, Germany) for 4 h to simulate natural abiotic weathering. Plastic was subsequently weighed and sterilized with 70% ethanol, and then washed with sterile water and set aside in a sterile hood until dry.

#### 4.2.2. Growth of PE-Degrading Bacteria

Consortium strains were grown on low-salt lysogeny broth (LB) agar plates (10 g/L tryptone, 5 g/L yeast extract, 5 g/L NaCl, 15 g/L agar, pH 7.0) at 30 °C. Strains were then grown in liquid LB to mid-exponential phase and normalized to an OD_600_ value of 1.000 to standardize bacterial counts using a BioSpec mini−1240 UV-Vis Spectrophotometer (Shimadzu Scientific Instruments, Columbia, MD, USA). After normalization, to monitor growth on PE consortia bacteria were spun down to a pellet and washed twice with a modified Liquid Carbon Free Basal Medium (LCFBM) adapted from the American Society for Testing and Materials (ASTM) standard for studying the bacteria resistance to plastics (ASTM G22−76) (per liter: 0.05% (wt/vol) yeast extract, 0.2% (wt/vol) (NH_4_)_2_ SO_4_, and 1% (vol/vol) trace elements (0.1% [wt/vol] FeSO_4_·7 H_2_O, 0.1% [wt/vol] MgSO_4_·7 H_2_O, 0.01% [wt/vol], CuSO_4_·5 H_2_O, 0.01% [wt/vol] MnSO_4_·5 H_2_O, and 0.01% [wt/vol] ZnSO_4_·7 H_2_O) in 10 mM pH 7.4 sodium phosphate buffer.). Cultures were inoculated by the addition of 100 µL of each rinsed isolate to 50 mL of 0.2% *w*/*v* PE-supplemented LCFBM in a 250 mL culture flask. Samples were incubated statically to facilitate biofilm formation [38] at 30 °C for 30 days.

#### 4.2.3. Growth Conditions of E. coli Strains for Gibson Assembly, Plasmid Isolation, and Protein Overexpression

*E. coli* DH5α bacteria were grown at 37 °C. Overnight cultures of these strains were grown in LB media, shaking at 180 rpm. During transformation of the pMTBN28 cyp vector (Supplemental Text S1), room temperature super optimal broth with a proprietary (NEB, Ipswich, MA, USA) catabolite repression (SOC) media (2% vegetable peptone, 0.5% yeast extract, 10 mM NaCl, 2.5 mM KCl, 10 mM MgCl2, 10 mM MgSO_4_, 20 mM glucose, pH 7.0) was used to recover *E. coli* cells after Gibson Assembly. *E. coli* transformants were grown on and selected from using LB agar plates supplemented with 50 µg/mL kanamycin at 37 °C. For protein overexpression, *E. coli* BL21 (DE3) and C41 (DE3) cell lines containing the pMTBN28 cyp plasmid were grown shaking in Terrific Broth (TB) media (12 g/L tryptone, 24 g/L yeast extract, 4 mL/L glycerol, 0.017 M KH2 PO4, 0.072 M K2 HPO4, 50 µg/mL kanamycin), 0.5 mM δ-aminolaevulinic acid (5-ALA) and 0.4 mM IPTG were added to induce protein expression before purification of CYP102 A5 protein.

### 4.3. Measurement of Bacterial Growth for Evaluating PE Degradation

After microbial growth in LCFBM cultures, aliquots of culture were serially diluted in triplicate to obtain the CFU/mL every 5 days. CFU/mL values were averaged across three separate flasks. CFU mL values for the control cultures (LCFBM only samples) were collected to ensure no bacterial contamination occurred. The culture pH values were measured using a pH probe and verified with pH strips having a range of 5.1−7.2 (VWR, Tualatin, OR, USA).

### 4.4. Weight Loss of PE Plastic and Water Absorbance Changes in Bacterial Culture

Following 30 days of bacterial biodegradation, the weight of PE powder and film was measured using the method described in Hadar, Y., and A. Sivan., 2004 [38]. Plastic was separated from the media using vacuum filtration with Watman #6 filter paper to ensure efficient recovery. The plastic film and powder were washed for 4 h using 2% SDS to remove bacteria from the plastic surface [74,75]. The surfactant and plastic were separated using gravity filtration and subsequently washed with sterile distilled water and 70% ethanol to remove any cell debris or residual bacteria colonized on the plastic surface. The plastic was then dried at 50 °C in a hot air oven to evaporate any remaining water. The percent weight reduction was calculated using the formula described by Kyaw et al., 2014 [76].

(1) $$\%\;weight\;reduction=\frac{\left(initial\;weight-final\;weight\right)}{initial\;weight\;}\times 100$$

For water absorbance calculations, the initial mass (M_1_) was analyzed after 30 days of incubation with the bacterial consortium alongside a comparative control of plastic inoculated in LCFBM for 30 days without bacteria following the sterilization and cleaning of the remaining plastic film and particles, ensuring complete removal of surfactant that may interfere with water absorbance. 50 mL of sterile distilled water was then added to each culture bottle. All flasks were then incubated at 30 °C for 5 days, and the average mass (M_2_) was determined after 120 h. The percentage of water absorption coefficient (a%) was calculated using the formula:

(2) $$a\%=\frac{\left(M_{2}\;-\;M_{1}\right)}{M_{1}}\times 100$$

### 4.5. SEM Analysis of Bacteria-Degraded PE Plastic

After 30 days of culture growth, PE sheets and powder were analyzed using SEM imaging. After weight loss measurements, the surface landscape of PE films and PE powder was examined using a scanning electron microscope (SEM). Both PE powder and PE film samples were dried before imaging and were handled identically, ensuring that any observed surface alterations could be attributed to biological activity rather than sample processing. A 4 nm carbon coating was added to both samples, and images were taken with a 1 μs dwell time. SEM was performed at the Multi-Scale Microscopy Core (MMC) with technical support from the Oregon Health and Science University (OHSU)/FEI Living Lab and the OHSU Center for Spatial Systems Biomedicine (OCSSB).

### 4.6. FTIR Analysis of Bacteria-Degraded PE Plastic

ATR-FTIR analysis was used to analyze the effects of biodegradation. Molecular changes in the PE plastic sheets before and after biodegradation by the bacterial consortium were measured using a Thermo iS5 FTIR Spectrometer with iD7 ATR accessory (Thermo-Fischer, Waltham, MA, USA). Samples were scanned at a resolution of 4 cm^−1^ between 600 cm^−1^ and 4000 cm^−1^. The relative absorbance intensities of the carbonyl index, ester carbonyl bond, keto carbonyl bond, terminal double bond (vinyl), and the internal double bond to that of the methylene bond were calculated using the following formulas, described below. I refers to a particular wavenumber of interest (cm^−1^) for PE polymers [77,78]. Molecular indexes (I) were determined as a ratio to the control wavenumber (cm^−1^) 1462 for the following: Keto-Carbonyl Index (KBI) (1715); Ester Carbonyl Bond Index (ECBI) (1740); Vinyl Bond Index (VBI) (1650); Internal Double Bond Index (IDBI) (908); and Keto-Carbonyl Index (KBI) (1714).

### 4.7. Detection of PE Degradation Products

After 30 days of biodegradation of PE by the full consortium of bacteria in LCFBM, the culture medium was collected and centrifuged for 15 min at 10,000 rpm at 4 °C to collect the supernatant. The supernatants from this culture and an identical sample containing LCFBM, PE, and no bacteria as the control were extracted with an equal volume of ethyl acetate (Sigma Aldrich, St. Louis, MO, USA) and subsequently concentrated utilizing a rotovap. The product was resuspended in 1 mL of ethyl acetate and filtered with a 0.22 μm filter. Products were analyzed using a 7890 A GC and a 5975 C MSD for gas chromatography–mass spectroscopy on electron impact ionization mode (Agilent, Santa Clara, CA, USA). The metabolites were separated on a Restek Rxi−5 Sil MS column (30 m × 0.25 mm × 0.25 μm, Restek, Center County, PA, USA). The oven temperature was initially set at 40 °C for 3 min, then increased to 280 °C at 10 °C/min, and was maintained at 280 °C for 4 min. For the detector, an electron ionization mode was set at 230 °C. The temperature of the detector and the injector was set to 150 °C. Sample volumes of 1 μm were taken per injection with a split ratio of 1:10. Samples were scanned at a range of 34−500 amu. Peak assignment was based on a comparison with the mass spectral database, W8 N08.L (Hewlett-Packard, Palo Alto, CA, USA).

### 4.8. Gene of Interest Identification and Expression in PE Plastic Conditions

Genes associated with PE degradation were identified using the Plastic Biodegradation Database—PlasticDB [31]. Genes to investigate by RT-qPCR were selected based on calculated sequence identity according to the NCBI Protein BLAST algorithm and Jalview Clustal Omega Version 2 [79] multiple sequence alignment. Primers were designed utilizing the Primer-BLAST tool and are listed in Table S3.

Cultures were harvested after the log phase was reached for PE cultures and the controls, respectively. Whole cellular cultures were first spun and lysed to isolate RNA using the RNeasy kit (Qiagen, Germantown, MD, USA) according to manufacturer instructions. Reverse transcription of cDNA was performed according to manufacturer recommendations using the High-Capacity cDNA Reverse Transcription Kit (Thermo Fisher, Waltham, MA, USA). Pure cDNA, along with designed primers (Thermo Fisher, Waltham, MA, USA) with the iTaq Universal SYBR Green supermix (Bio Rad, Hercules, CA, USA), was amplified on a CFX thermal cycler (BioRad, Hercules, CA, USA). All 16 S ribosomal genes of each isolate were used as housekeeping genes in both individual isolate and full consortium cDNA samples. Controls with no template DNA added (*n* = 3), along with a negative control of pure water, were included in each run. After assembly of PCR samples in triplicate, the samples were run according to the manufacturer’s instructions. Mean cycle threshold values (C_t_) were estimated along with melting curve analysis to confirm PCR primer and product specificity. Each cDNA sample was run against a standard curve of concentrations, and the primer efficiency was determined after generation of a 5-point standard curve. Changes in gene expression were calculated by the Pfaffl method, where GOI refers to the Gene of Interest and HKG refers to the Housekeeping Gene.

(3) $$\frac{{\left(E_{GOI}\right)}^{\Delta Ct\;GOI}}{{\left(E_{HKG}\right)}^{\Delta Ct\;HKG}}$$

### 4.9. Expression and Purification of CYP102 A5

Gibson Assembly was performed (Supplementary Information S1) to generate the pMT28 BNcyp plasmid encoding the CYP102 A5 protein sequence using primers in Table S4. For expression of CYP102 A5, sterile LB (60 mL) containing 50 μg/mL of kanamycin was inoculated with *E.coli* Overexpress ™ C41 (DE3) containing the plasmid and grown overnight at 37 °C, shaking at 200 rpm. Saturated overnight cultures were diluted 1:100 in 1 L of TB containing 50 μg/mL kanamycin. Cultures continued shaking under the same conditions until the OD_600_ was 0.4, and 0.5 mM δ aminolaevulinic acid was added immediately to the culture. Cultures continued shaking until an OD_600_ of 0.8 was reached, and 0.4 mM IPTG was added to chilled flasks. The cultures were left to grow and overexpress for 18 h at 30 °C and shaking at 100 rpm. Bacterial cultures were harvested by centrifugation at 5000× *g* for 25 min at 4 °C, and cell pellets were stored at −80 °C after being flash frozen in LN_2_.

For purification, pellets were resuspended in 10 mL lysis buffer per gram of pellet (50 mM KCl, 100 mM potassium phosphate buffer, pH 7.4) with the following additions: 0.5 mM PMSF, 1 mM Benzamidine, 1 µg/mL Leupeptin, 0.7 µg/mL Pepstatin, 0.01 mg/mL DNase, 1 mM MgCl2, 0.2 mg/mL lysozyme, and 1 mM TCEP. Pellets were gently homogenized and then lysed on a stir plate for 1 h at 4 °C. Lysed cells were sonicated for three total rounds of 6 repeats of 20 sec on (~70% power), 20 sec off, followed by 1 min rest between the individual rounds. The sonicated lysate was subsequently centrifuged (40,000× *g*, 35 min, 4 °C) to remove the cell debris. Ni-NTA resin equilibrated in lysis buffer containing 5 mM I midazole (Takara Bio, San Jose, CA, USA) was left to batch bind with the clarified supernatant while rotating for 1 h at 4 °C. Afterwards, the resin was spun down at 900× *g* at 4 °C for 5 min and nd packed in a column under gravity. After collecting the flow through, the Ni-NTA resin was washed with a stepwise imidazole gradient (5−40 mM imidazole) and then CYP102 A5 was eluted with 250 mM imidazole buffer using a peristaltic pump. Wash and elution fractions were analyzed using a Nanodrop ND−1000 Spectrophotometer (Thermo Fisher, Waltham, MA, USA) and by separation using SDS-PAGE. Elutions were concentrated to ~2–3 mg/mL in an Amicon Ultra 15 50 kDa cutoff concentrator (Sigma Aldrich, Watham, MA, USA), and the resulting concentrate (~3 mL) was left to dialyze overnight using 12,000–14,000 MWCO tubing (Spectra Labs, Rancho Dominguez, CA, USA). Samples were concentrated further to approximately 10 mg/mL and filtered through 0.45 μm centrifugal filter, spun at 8000 rpm for 1 min at 4 °C. The filtered sample was manually injected onto an AKTA Pure 150 FPLC system (Cytiva, Marlborough, MA, USA) equipped with a size exclusion chromatographic (SEC) Superdex 200 10/300 (Cytiva, Marlborough, MA, USA) column. SEC fractions were resolved on an SDS-PAGE gel. Fractions containing pure CYP102 A5 were combined and concentrated using a 50 kDa concentrator to 3 mg/mL and flash frozen in liquid nitrogen before storing at −80 °C

### 4.10. NADPH Consumption Assays of CYP102 A5

NADPH consumption was used to determine if CYP102 A5 is active in the presence of substrate. A baseline test for one of many native substrates of CYP102 A5, palmitic acid, was used to establish activity (Supplementary Information S2). In brief, in a 1 mL reaction volume, 50 mM of a 5 mM stock of palmitic acid in ethanol, 55 nM of purified CYP102 A5, and 200 nM of NADPH were added in a 1 mL total reaction volume suspended in buffer (50 mM pH 7.4 potassium phosphate, 100 mM potassium chloride). Compounds were added in order listed, and there was 30 s between the addition of each component. After the 200 nM of NADPH was added to the quartz cuvette, the cuvette was immediately added to an Agilent Cary 60 UV-Vis (Agilent, Santa Clara, CA, USA). The absorbance at 340 nm was collected every single second for 1 min. The beam mode was set to dual beam, and the data interval was 2.00 nm. To establish the activity of CYP102 A5 in the presence of PE powder, 10 mg/mL of PE powder was pre-added to a quartz cuvette. The total 1 mL reaction volume consists of 400 nM CYP102 A5, 100 nM NADPH, and the same buffer as listed above. NADPH was homogenized in buffer before addition to the quartz cuvette. Careful effort was focused on ensuring no plastic was stuck above the buffer line. Subsequently, 400 nM of CYP102 A5 was spiked into the sample and left to equilibrate for 10 s before collecting UV-Vis spectra. After the 10 s had elapsed, recording was started on an Agilent Cary 60 UV-Vis (Agilent, Santa Clara, CA, USA). The absorbance at 340 nm was collected every second for 60 s in total. The beam mode was set to dual beam, and the data interval was 2.00 nm.

### 4.11. Long-Term NADPH-Regeneration Assay and FTIR Analysis of PE Plastic After Treatment with CYP102 A5

Briefly, 10 mg/mL of PE plastic, 10 mM glucose 6-phosphate, 1 unit/mL of glucose 6-phosphate dehydrogenase, 0.5 mM NADPH, and 400 nM of CYP102 A5 (or without CYP102 A5 for the hydrolysis control) were left shaking for 18 hrs at 37 °C in buffer (50 mM potassium phosphate, 100 mM KCl). After 18 h had elapsed, plastic was filtered from the buffer via gravity using Whatman #1 filter paper. PE was washed 3× with a 10:1 H_2_O: methanol solution and then washed 3 more times with 2% SDS. The plastic was left to dry overnight in a 60 °C oven before ATR-FTIR analysis. Molecular changes in the PE plastic sheets before and after biodegradation by the CYP102 A5 were measured using a Thermo iS5 FTIR Spectrometer with iD7 ATR accessory (Thermo-Fischer, Waltham, MA, USA). Samples were scanned at a resolution of 4 cm^−1^ between 600 cm^−1^ and 4000 cm^−1^.

## Figures and Tables

**Table 1:** ATR-FTIR analysis of powdered and film LDPE ^1^.

Sample Type	Carbonyl Bond Index (I1714/I1463)	Keto Carbonyl Bond Index (I1715/I1465)	Internal Double Bond Index (I908/I1465)	Ester Carbonyl Index (I1740/I1465)	Vinyl Bond Index (I1650/I1465)
Untreated Powder	0.12 ± 0.01	0.12 ± 0.01	0.18 ± 0.01	0.13 ± 0.01	0.13 ± 0.01
Treated Powder	0.16 ± 0.03	0.16 ± 0.03	0.22 ± 0.03	0.17 ± 0.03	0.16 ± 0.03
Untreated Film	0.08 ± 0.00	0.15 ± 0.01	0.10 ± 0.00	0.09 ± 0.00	0.12 ± 0.03
Treated Film	0.16 ± 0.02	0.27 ± 0.02	0.12 ± 0.02	0.17 ± 0.02	0.11 ± 0.01

^1^ Molecular index and confidence intervals were calculated by relative intensities of vibrational bands of interest from ATR-FTIR analysis of film and powdered LDPE incubated in carbon-free medium with bacteria for 4 weeks; *n* = 6.

**Table 2:** Molecular index and confidence intervals calculated by relative intensities of vibrational bands of interest from ATR-FTIR analysis of powdered LDPE incubated with CYP102 A5 and a NADPH regenerating system for 18 h ^1^.

Sample Type	Carbonyl Bond Index (I_1714_/I_1463_)	Keto Carbonyl Bond Index (I_1715_/I_1465_)	Internal Double Bond Index (I_908_/I_1465_)	Ester Carbonyl Index (I_1740_/I_1465_)	Vinyl Bond Index (I_1650_/I_1465_)
Control powdered PE	0.10 ± 0.02	0.12 ± 0.02	0.17 ± 0.02	0.15 ± 0.02	0.18 ± 0.01
CYP102 A5, powdered PE	0.14 ± 0.01	0.15 ± 0.01	0.18 ± 0.01	0.16 ± 0.01	0.19 ± 0.01

^1^ *n* = 6.

## Data Availability

The data are contained within the article.

## Supplementary Materials

The following supporting information can be downloaded at: https://www.mdpi.com/article/10.3390/ijms26188775/s1. Reference [80] has been mentioned in the text.

## Abbreviations

The following abbreviations are used in this manuscript:

PE	polyethylene
LDPE	low-density polyethylene
ATR-FTIR	attenuated total reflectance–Fourier transform infrared spectroscopy
GC/MS	gas chromatography–mass spectrometry
LCFBM	liquid carbon free basal medium
CFU	colony forming units
