# Supplementary material for: A Cytochrome P450 Facilitates Polyethylene Metabolism in a Microbial Community
Source: Int J Mol Sci. 2025 Sep 9;26(18):8775. doi: 10.3390/ijms26188775 (PMC12470125; doi:10.3390/ijms26188775)
Supplement: Supplementary file 1 [file ijms-26-08775-s001.zip › ijms-3808576-supplementary.pdf]

Table of Contents

1. Supplemental Texts
    - *a.* **S1.** Cloning of CYP102A5 from *B. thuringiensis* C15 and expression in *E.coli*
    - *b.* **S2.** Amino acid sequences of CYP102A5 registered on the Cytochrome P450 website (https://drnelson.uthsc.edu/), CYP102A5.v1 from Yeom et. al (2020), and CYP102A5.v2
    - *c.* **S3.** Description of alignments of the CYP102A5 variants
2. Supplementary Tables
    - a. **Table S1.** Degradative products identified after degradation of PE film by the bacterial consortium
    - b. **Table S2.** Degradative products identified after degradation of PE powder by the bacterial consortium
    - c. **Table S3.** Primer sequences utilized for RT-qPCR genes of interest and 16S RNA genes
    - d. **Table S4.** Primer sequences utilized for Gibson Assembly of CYP102A5 into the pET28b(+) expression vector
3. **3.** Supplementary Figures
    - a. **Figure S1.** Individual strains and consortia growth on PE film in LCFBM
    - b. **Figure S2.** qRT-PCR of the genes of interest when grown in the consortium
    - c. **Figure S3.** Purification of CYP102A5.v2
    - d. **Figure S4.** NADPH consumption of PE in the presence of palmitic acid
    - e. **Figure S5.** Clustal Omega alignment of CYP102A5, CYP102A5.v1, and CYP102A5.v2
4. References

1. Supplemental Texts

S1. Gibson Assembly cloning of CYP102A5 from *B. thuringiensis* C15 and expression in *E.coli*

Sequence information was obtained via referencing the NIH BioProject (PRJNA517285) database (https://www.ncbi.nlm.nih.gov/bioproject/) . Using NEBuilder (https://nebuilder.neb.com), flanking primers were designed to amplify the CYP102A5 gene (3255bp) from *B. thurigenesis* C15 (Strain 9.1). The primers (**Table S4**) included partial overlapping sequences to facilitate assembly to the protein overexpression vector pET28b(+). Primer validation was conducted using the Benchling (www.benchling.com) and the NIH Open Reading Frame Finder (https://www.ncbi.nlm.nih.gov/orffinder/) programs to validate proper protein translation.

To amplify the CYP102A5 gene, genomic DNA was isolated from isolate 9.1 using a column-based isolation kit (QUIAGEN, Germantown, MD). PCR was subsequently performed in 25µL reactions containing 200µM dNTPs, 0.5µM upstream and downstream primers, 2µL 100ng DNA template, 0.02U/µL Q5- High-Fidelity DNA polymerase, and 1X Q5 High GC Enhancer in 1X Q5 Reaction Buffer (New England Biolabs, Ipswich, MA). A Bio-Rad T100 Thermocycler (Bio-Rad, Hercules, CA) was programmed with a touchdown PCR protocol according to manufacturer recommendations. Fragments were confirmed by DNA agarose gel electrophoresis and visible bands extracted and purified using a gel extraction kit for future Gibson Assembly (QUIAGEN, Hilden, DE). The pET28b(+) plasmid (Novagen, Madison, WI) was linearized in the multiple cloning site by a double restriction digest with BamHI-HF (New England Biolabs, Ipswich, MA) and NotI-HF (New England Biolabs, Ipswich, MA). Digest occurred in a 50µL reaction containing 1ug of unmodified pET28b(+), 1X rCutSmart Buffer (New England Biolabs, Ipswich, MA), and 20 units of both BamHI-HF and NotI-HF. The reaction was incubated at 37°C in a Bio-Rad T100 Thermocycler (Bio-Rad, Hercules,

CA) for 60 minutes. Parent plasmid pET28b(+)digestion was verified using DNA agarose gel electrophoresis.

Gibson Assembly was performed using a Gibson Assembly Cloning Kit (New England Biolabs, Ipswich, MA) following the procedure described in Figure 10. In brief, reaction mixtures containing a Gibson Assembly master mix and a 5:1 molar ratio of inserts to linearized plasmid (0.02 pmol vector: 0.106 pmol insert) were incubated at 50°C for 60 minutes. Completed Gibson Assembly reaction mixtures were stored at -20°C until transformation. Transformation was performed with chemically competent *E. coli* DH5α or 10-β (New England Biolabs, Ipswich, MA). After confirming the construct to be correct by DNA sequencing, the resulting plasmid pMTBN28cyp was transformed into chemically competent *E.coli* BL21(DE3) (New England Biolabs, Ipswich, MA) for protein expression.

S2. Sequence of CYP102A5 variants

The 1066 amino acid sequence of CYP102A5 from *Bacillus cereus* registered on the Cytochrome P450 website (https://drnelson.uthsc.edu/), and on UniProt under the number Q81BF4 reads as follows:

MEKKVSAIPQPKTYGPLGNLPLIDKDKPTLSFIKIAEEYGPIFQIQTLSDTIIVVSG
HELVAEVCDETRFDKSIEGALAKVRAFAGDGLFTSETHEPNWKKAHNILMPTFSQRA
MKDYHAMMVDIAVQLVQKWARLNPNENVDVPEDMTRLTLDTIGLCGFNYRFNSF
YRETPHPFITSMTRALDEAMHQLQRLDIEDKLMWRTKRQFQHDIQSMFSLVDNIIAE
RKSSGDQEENDLLSRMLNVPDPETGEKLDDENIRFQIITFLIAGHETTSGLLSFAIYFLL
KNPDKLKKAYEEVDRVLTDPTPTYQQVMKLKYMRMILNESLRLWPTAPAFSLYAKE
DTVIGGKYPIKKGEDRISVLIPQLHRDKDAWGDNVEEFQPERFEELDKVPHHAYKPF
GNGQRACIGMQFALHEATLVMGMLLQHFELIDYQNYQLDVKQTLTLKPGDFKIRIL
PRKQTISHPTVLAPTEDKLKNDEIKQHVQKTPSIIGADNLSLLVLYGSDTGVAEGIARE
LADTASLEGVQTEVVALNDRIGSLPKEGAVLIVTSSYNGKPPSNAGQFVQWLEELKP

DELKGVQYAVFGCGDHNWASTYQRIPRYIDEQMAQKGATRFSKRGEADASGDFEE
QLEQWKQNMWSDAMKAFGLELNKNMEKERSTLSLQFVSRLGGSPLARTYEAVYASI
LENRELQSSSSDRSTRHIEVSLPEGATYKEGDHLGVLPVNSEKNINRILKRFGLNGKD
QVILSASGRSINHIPLDSPVSLLALLSYSVEVQEAATRAQIREMVTFTACPPHKKELEAL
LEEGVYHEQILKKRISMLDLLEKYEACEIRFERFLELLPALKPRYYSISSSPLVAHNRLSI
TVGVVNAPAWSGEGTYEGVASNYLAQRHNKDEIICFIRTPQSNFELPKDPETPIIMVG
PGTGIAPFRGFLQARRVQKQKGMNLGQAHLYFGCRHPEKDYLYRTELENDERDGLI
SLHTAFSRLEGHPKTYVQHLIKQDRINLISLLDNGAHLYICGDGSKMAPDVEDTLCQ
AYQEIHEVSEQEARNWLDRVQDEGRYGKDVWAGI

The 1065 amino acid sequence of CYP102A5.v1 from Yeom *et. al.,* (2020) appears below:

MEKKVSAIPQPKTYGPLGNLPLIDKDKPTLSFIKIAEEYGPIFQIQTLSDTIIVVSG
HELVAEVCDETRFDKSIEGALAKVRAFAGDGLFTSETHEPNWKKAHNILMPTFSQRA
MKDYHAMMVDIAVQLVQKWARLNPNENVDVPEDMTRLTLDTIGLCGFNYRFNSF
YRETPHPFITSMTRALDEAMHQLQRLDIEDKLMWRTKRQFQHDIQSMFSLVDNIIAE
RKSSGDQEENDLLSRMLNVPDPETGEKLDDENIRFQIITFLIAGHETTSGLLSFAIYFLL
KNPDKLKKAYEEVDRVLTDPTPTYQQVMKLKYIRMILNESLRLWPTAPAFSLYAKED
TVIGGKYPIKKGEDRISVLIPQLHRDKDAWGDNVEEFQPERFEELDKVPHHAYKPFG
NGQRACIGMQFALHEATLVMGMLLQHFELIDYQNYQLDVKQTLTLKPGDFKIRILP
RKQTISHPTVLAPTEDKLKNDEIKQHVQKTPSIIGADNLSLLVLYGSDTGVAEGIAREL
ADTASLEGVQTEVVALNDRIGSLPKEGAVLIVTSSYNGKPPSNAGQFVQWLEELKPD
ELKGVQYAVFGCGDHNWASTYQRIPRYIDEQMAQKGATRFSKRGEADASGDFEEQL
EQWKQNMWSDAMKAFGLELNKNMEKERSTLSLQFVSRLGGSPLARTYEAVYASILE
NRELQSSSSDRSTRHIEVSLPEGATYKEGDHLGVLPVNSEKNINRILKRFGLNGKDQVI
LSASGRSINHIPLDSPVSLLDLLSYSVEVQEAATRAQIREMVTFTACPPHKKELEALLEE
GVYHEQILKKRISMLDLLEKYEACEIRFERFLELLPALKPRYYSISSSPLVAHNRLSITVG

VVNAPAWSGEGTYEGVASNYLAQRHNKDEIICFIRTPQSNFELPKDPETPIIMVGPGT
GIAPFRGFLQARRVQKQKGMNLGQAHLYFGCRHPEKDYLYRTELENDERDGLISLH
TAFSRLEGHPKTYVQHLIKQDRINLISLLDNGAHLYICGDGSKMAPDVEDTLCQAYQ
EIHEVSEQEARNWLNRVQDEGRYGKDVWAGI

The 1065 amino acid sequence of CYP102A5.v2 can be found on the NIH BioProject PRJNA517285 under the assembly GCF_039954905.1 under "bifunctional P450/NADPH—P450 reductase" under the symbol name *cyp*D. The protein code is WP_074555304.1. The amino acid sequence is as follows:

MEKKVSAIPQPKTYGPLGNLPLIDKDKPTLSFIKIAEEYGPIFQIQTLSDTIIVVSGHELVAEVC
DETRFDKSIEGALAKVRAFAGDGLFTSETHEPNWKKAHNILMPTFSQRAMKDYHAMMVDIAVQL
VQKWARLNPNENVDVPEDMTRLTLDTIGLCGFNYRFNSFYRETPHPFITSMTRALDEAMHQLQR
LDIEDKLMWRTKRQFQHDIQSMFSLVDNIIAERKSNGNQEENDLLSRMLNVPDPETGEKLDDENI
RFQIITFLIAGHETTSGLLSFAIYFLLKNPDKLKKAYEEVDRVLTDPTPTYQQVMKLKYIRMILNESLRL
WPTAPAFSLYAKEDTVIGGKYPIKKGEDRISVLIPQLHRDKDAWGDNVEEFQPERFEELDKVPHHA
YKPFGNGQRACIGMQFALHEATLVMGMLLQHFELIDYQNYQLDVKQTLTLKPGDFKIRILPRKQTI
SHPTVLASTEDKLKNDEIKQHVQKTPSIIGADNLSLLVLYGSDTGVAEGIARELADTASLEGVQTEV
VALNDRIGSLPKEGAVLIVTSSYNGKPPSNAGQFVQWLEELKPDELKGVQYAVFGCGDHNWAST
YQRIPRYIDEQMAQKGATRFSKRGEADASGDFEEQLEQWKQNMWSDAMKAFGLELNKNMEKE
RSTLSLQFVSRLGGSPLARIYEAVYASILENRELQSSSSDRSTRHIEVSLPEGATYKEGDHLGVLPVNS
EKNINRILKRFGLNGKDQVILSASGRSINHIPLDSPVSLLDLLSYSVEVQEAATRAQIREMVTFTACP
PHKKELEALLEEGVYHEQILKKRISMLDLLERYEACEIRFERFLELLPALKPRYYSISSSPLVAHNRLSIT
VGVVNAPAWSGEGTYEGVASNYLAQRHNKDEIICFIRTPQSNFELPKDPETPIIMVGPGTGIAPFR
GFLQARRVQKQKGINLGQAHLYFGCRHPEKDYLYRTELENDERDGLISLHTAFSRLEGHPKTYVQH
LIKQDRINLISLLDNGAHLYICGDGSKMAPDVEDTLCQAYQEIHEVSEQEARNWLDRVQDEGRYG
KDVWAGI

S3. Alignments of CYP102A5.v2 to other variants

CYP102A5.v2 was compared to other variants in the literature. Calculated alignments were done on NCBI protein blast with the query sequence of CYP102A5 from *Bacillus cereus*. The percent sequence identity was found to be 99.25%, the query cover was 100%, and the E-value was 0. Furthermore, CYP102A5.v2 was compared to CYP102A5.v1, a published PE-degrading enzyme. BlastP alignment identified a 99.34% sequence identity between these two variants with a query cover of 100%, and a E-value of 0.

2. Supplementary Tables

Table S1. Degradative products produced after bacterial biodegradation of LDPE film[1]

RT	Name	% Similarity	Molecular Weight (g/mol)	Chemical Formula
3.36	Butane, 1-ethoxy-	91	102.1748	$C_6H_{14}O$
3.912	Hexane, 2,5-dimethyl-	95	114.23	C_8H_{18}
4.227	Pentane, 2,3,4-trimethyl-	81	114.2285	C_8H_{18}
27.359	Octadecane, 1-isocyanato-	79	295.5032	$C_{19}H_{37}NO$

[1]Peak list of compounds found after biodegradation with PE film.

Table S2. Degradative products after bacterial degradation of powdered PE plastic

RT	Name	% Similarity	Molecular weight (g/mol)	Chemical Formula
3.349	Butane, 1-ethoxy-	91	102.1748	$C_6H_{14}O$
3.912	Hexane, 2,5-Dimethyl	93	114.23	C_8H_{18}
8.494	Diethylene glycol[2]	83	106.1204	$C_4H_{10}O_3$
17.777	Hexadecane	93	226.44	$C_{16}H_{34}$
19.186	Azulene, 1,4-dimethyl-7-(1-methylethyl) [2]	87	198.30	$C_{15}H_{18}$
19.379,<br>19.486,<br>19.582,<br>19.577,<br>19.856	Several unidentified compounds	n/a	n/a	n/a
21.266	Methyl palmitate	81	270.4507	$C_{17}H_{34}O_2$
21.66	Palmitic Acid	83	256.43	$C_{16}H_{32}O_2$
22.916<br>23.79	Unknown large phenol ring compound [2]	n/a	n/a	n/a
23.286	1-Triacontanol	91	438.81	$C_{30}H_{62}O$
24.728	Tricosane	91	324.63	$C_{23}H_{48}$
25.558	Docosane	96	310.6027	$C_{22}H_{46}$
25.698	Unknown compound	n/a	n/a	n/a

25.826	2,4-Bis(1-phenylethyl)phenol[2]	81	302.4	$C_{22}H_{22}O$
26.207	Diethylene glycol dibenzoate[2]	86	314.3	$C_{18}H_{18}O_5$
26.609	Dioctyl phthalate [2]	91	390.6	$C_{24}H_{38}O_4$
27.118	n-Hexacosane	97	366.7	$C_{26}H_{54}$
27.933	n-Tetracosane	96	338.65	$C_{24}H_{50}$
28.87	n-Hexacosane	94	366.7	$C_{26}H_{54}$

[1]Peak list of compounds found after biodegradation with powdered PE.

[2] Indicates a compound associated with a plastic additive.

Table S3. Primers used for RT-qPCR[1]

**Primer Name**	T_m 50mM Na^+ (°C)	**Mw** (ug/µmole)	**Extinction Coefficient** (OD/µmmol)	**Sequence 5-′3**	**Determined** T_A (°C)
CypD (9.1) F	59	6,128	219.6	GGG CTA GTA CCT ACC AAC GG	57
CypD (9.1) R	64	6,031	214.3	GAG CTC CAA TCC AAA CGC CT	57
BVMO (9.2) F	63	6,103	217.1	CAA TCT GCT CAA GGC CGG TA	60
BVMO (9.2) R	63	6,159	218.0	CAT CTG GGT CCA GCG GTA AG	60

Glutathione Dehydrogenase (10) F	62	6,081	224.7	CAA CAC TGC CAA GGT CAA GC	61
Glutathione Dehydrogenase (10) R	66	6,125	209.5	CTT TCG GGT TGA CGC AAT CG	61
nlhH (13.2) F	61	6,208.1	228.4	GAG GGC TAC CTG CTG GAA AG	58.5
nlhH (13.2) R	61	6,085	205.1	TGC CCT TCA TCT AGC AGT GG	58.5
16s Ribosomal (9.1) F	61	6,022.9	208.3	TGA GCC GTT ACC TCA CCA AC	58.5
16s Ribosomal (9.1) R	61	6,103.0	217.1	TAA CAC GTG GGT AAC CTG CC	58.5
16s Ribosomal (9.2) F	62	6,121	229.1	ACG CGA AGA ACC TTA CCA GG	58
16s Ribosomal (9.2) R	63	6,000.9	215.9	CCC AAC ATC TCA CGA CAC GA	58
16s Ribosomal (10) F	61	6134.0	215.5	GTT TAC GGC GTG GAC TAC CA	59
16s Ribosomal (10) R	63	6,063	212.7	TCA ACC TGG GAA CTG CAT CC	59
16s Ribosomal (13.2) F	62	6,116.0	203.5	CCT GGT AAG GTT CTT CGC GT	59
16S Ribosomal	62	6,176.1	235.4	AGT ACG GCC	59

(13.2) R				GCA AGG TTA AA	

[1]Primers utilized for quantitative PCR analysis. Primers were generated using the NCBI primer design tool and products were standardized to be between 100-200 bp. Forward primers are indicated with "F" and Reverse primers are indicated with "R".

Table S4. Primers for Gibson Assembly[1]

Location (to the gene of interest)	Sequence (5′->3′)
Upstream	agcatgactggtggacagcaaatgggtcggGAAAAAAAGTATCTGCCATTCCTC
Downstream	cttttctacaaacccgaccatatactCGTGAGCTCGTGGTGGTGGTGGTGGTGACT

[1] Uppercase indicate annealing segments for PCR for amplifying the gene encoding CYP102A5.

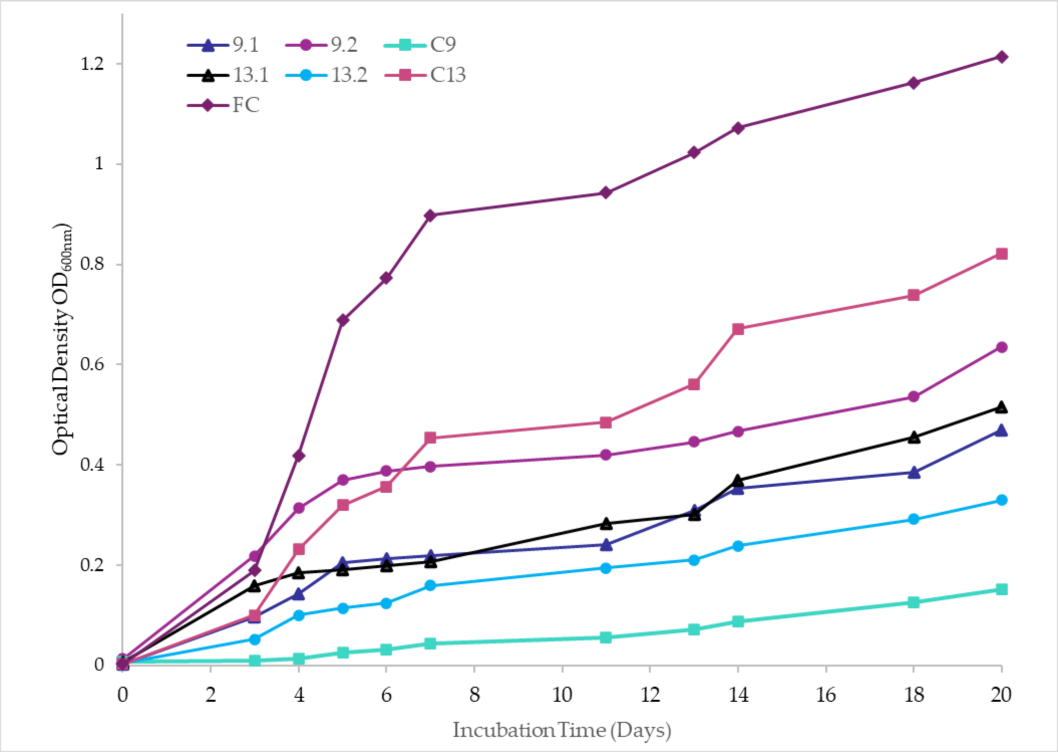

Figure S1. Growth of bacterial isolates in LCFBM using consumer PE plastic as a sole carbon source. Absorbance values from single isolates, consortium C9, consortium C13, and the full consortium containing all five strains. Growth occurred over 20 days in a liquid carbon-free base medium (LCFBM) with 0.2 w/v low-density polyethylene film. Cultures were normalized by OD_{600} to ensure equal amounts of bacteria added to all flasks, as described in Materials and Methods. Flasks were incubated statically at 30°C. Growth was measured every 48 hours blanked with LCFBM. Data presented are the averages of three replicate cultures for each isolate or consortium.

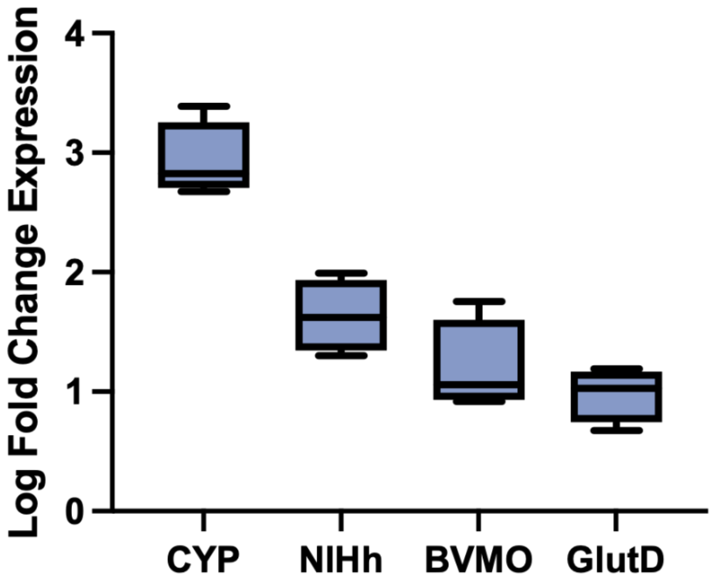

Figure S2. RT-qPCR expression data of genes of interest for the full consortium when grown on PE plastic. Log fold change expression determined by two-step quantitative real-time PCR (RT-qPCR) of genes putatively involved in PE biodegradation. Fold change expression was calculated (n=4) using the Pfaffl method for bacterial strains. Strains associated with each tested gene, and the associated primer sequences, are described in Supplementary Table 3. Log fold changes for genes in graph are as follows: CYP (2.929, p <0.0001), NlhH (1.634, p = 0.0012), BVMO (1.197, p = 0.3195), and *glutD* (0.981, p=0.0419).

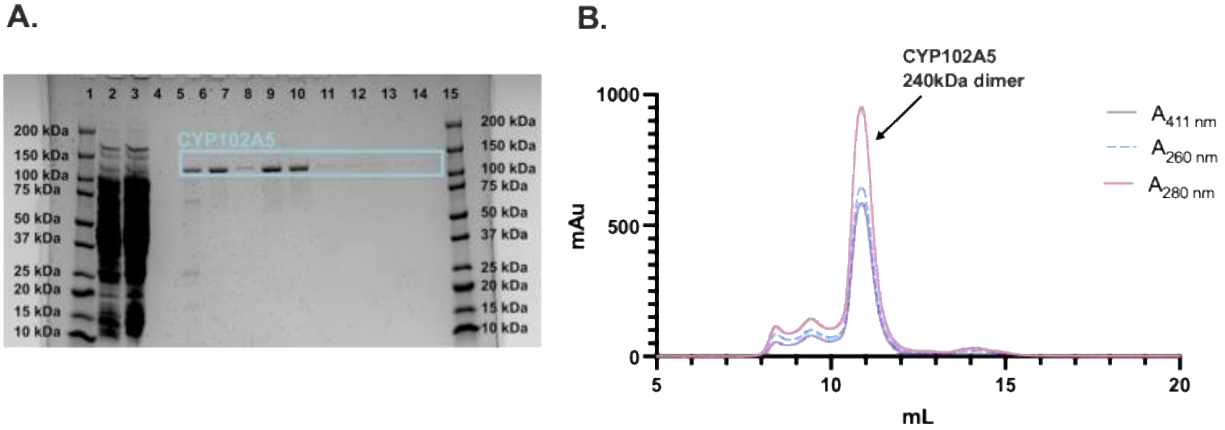

Figure S3. Purification of CYP102A5.v2 **(A)** Isolation of CYP102A5 via immobilized metal purification chromatography. Lane 1 is the protein ladder. Lane 2 is the

supernatant pre-batch binding. Lane 3 is the flow through. Lanes 4-6 are washes. Lanes 7-14 are elutions. Another ladder is added on lane 15. The gel image was annotated in Adobe Illustrator. **(B)** Chromatogram of CYP102A5 run on a AKÄTA FPLC system equipped with Superdex 200/10/300 column. The pink line is A_{280} nm, the tryptophan absorption peak. The dashed blue line, A_{260} is the DNA absorption peak. A_{411}, the purple peak is the absorption of the heme component. The peak attributable to CYP102A5 is annotated with an arrow and eluted at 10.9 ml. This figure was generated and graphed using Graph Pad Prism 10.

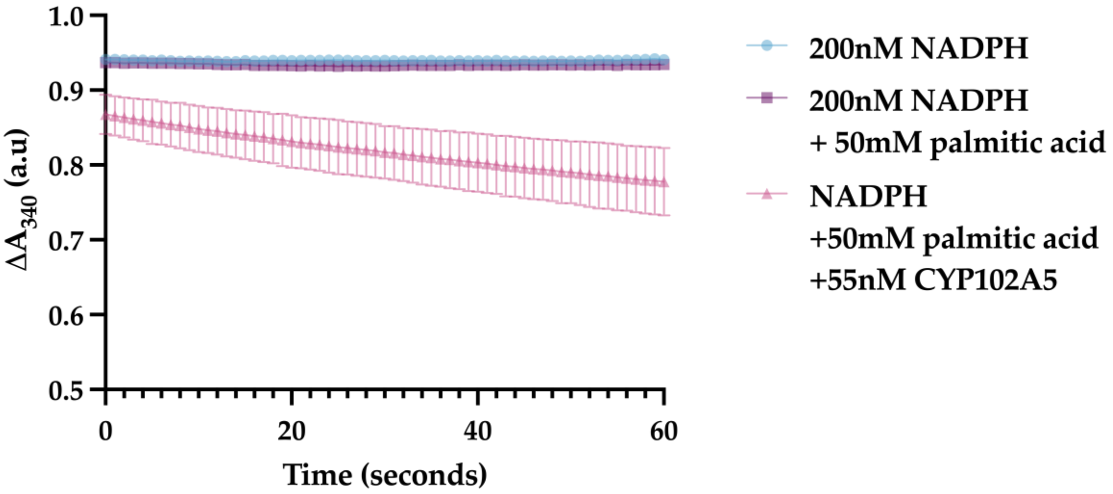

Figure S4. NADPH-consumption assay with palmitic acid as a substrate. Absorbance at 340nm in the first 60 seconds of CYP102A5.v2 incubation with 50mM palmitic acid (pink) (n=3) with 95% confidence intervals plotted. Controls with NADPH over 60 seconds (blue) and NADPH with substrate (purple) are included (n=3, error bars not shown). Scanning was done at 340cm^{-1}. The beam mode was set to dual beam and the data interval was 2.00nm. A_{340} values were collected every single second. Figure was generated using Graph Pad Prism 10.

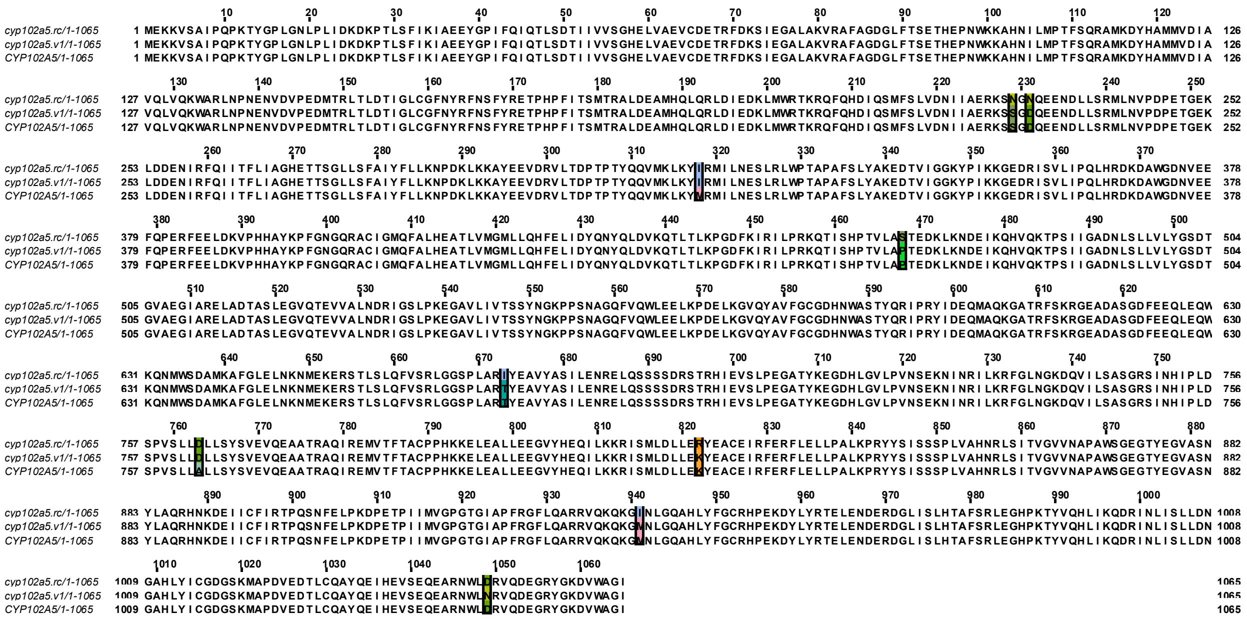

Figure S5. **Clustal Omega Alignment of CYP102A5 variants**. Clustal Omega alignment in the Jalview software of CYP102A5.v2 from this study to CYP102A5.v1 and CYP102A5. Colored areas indicate amino acid sequence changes colored with the Gecos Blossom color scheme from Kunzmann et. al (2020).

4. References

1. Yeom, S.-J., Le, T.-K., & Yun, C.-H. (2022). P450-driven plastic-degrading synthetic bacteria. *Trends in Biotechnology*, *40*(2), 166–179.

2. Kunzmann, P., Mayer, B.E. & Hamacher, K. Substitution matrix-based color schemes for sequence alignment visualization. *BMC Bioinformatics* **21**, 209 (2020). https://doi.org/10.1186/s12859-020-3526-6 and https://gecos.biotite-python.org/.
